# Single-port Laparoscopic Reversal of Hartmann's Procedure: Technique and Results

**DOI:** 10.1155/2011/356784

**Published:** 2011-06-16

**Authors:** Th. Carus, A. Emmert

**Affiliations:** Department of General, Visceral, and Vascular Surgery, Center of Minimally Invasive Surgery, Hospital Cuxhaven, 27474 Cuxhaven, Germany

## Abstract

In general, reversal of Hartmann's procedure is associated with a high morbidity and therefore leads to a low rate of intestinal restoration. Reversal of Hartmann's procedure has to be seen as a complex abdominal operation with the same possible complications as in other colorectal resections. By using the laparoscopic technique, operative access trauma by laparotomy can be minimized. After introducing single-port access into laparoscopic surgery beginning with cholecystectomies and sigmoid resections, we started with the first single-port laparoscopic reversal of Hartmann's procedure in January 2010. After excision of the colostoma, mobilization, and reponing into the abdominal cavity, the single-port trocar was placed at the stoma incision without any extra scar. We investigated whether the single-port laparoscopic reversal is as safely feasible as the “conventional” laparoscopic procedure. Till December 2010, single-port reversal operation was performed in 8 patients 2–4 months after Hartmann's procedure because of complicated diverticulitis. No conversion to “conventional” laparoscopic or open procedure was necessary in 1 patient one extra 5 mm trocar was used. The average operation time was 74 min. Except for one wound complication, the postoperative course was uncomplicated. The patients were discharged after 4 to 8 postoperative days. Single-port reversal of Hartmann's procedure has showed as a new method for minimizing the access trauma even further than “conventional” laparoscopic surgery.

## 1. Introduction

In earlier years, Hartmann's procedure has been the standard operation in the treatment of complicated sigmoid diverticulitis and of ileus due to obstruction of the left colon. Today most surgeons perform a single-stage procedure with a primary anastomosis—sometimes combined with a protective double-loop stoma. In patients with a complicated diverticulitis (sigmoid perforation and feculent peritonitis, Hinchey IV classification) Hartmann's procedure still has its place in modern surgical therapy.

Only few surgical departments perform the laparoscopical reversal of Hartmann's procedures, almost no department in single-port technique. In this retrospective study, we want to show our new technique with the aim to further minimize the access trauma.

## 2. Patients and Methods

In 2010, there were in total 147 colorectal resections in our department, and in 12 (8,2%) patients, we performed Hartmann's procedure (5 laparoscopic, 3 open) due to complicated diverticulitis. In 8 patients we performed an elective laparoscopical reversal of Hartmann's procedure in single-port technique.

### 2.1. Preoperative Treatment

Elective operation was performed 2–4 months after Hartmann's procedure. Preoperatively we examined the afferent loop and the rectal stump by endoscopy and contrast enema. One day before operation the patients had a bowel cleaning by oral intake of bisacodyl (Prepacol). On the day of surgery, a rectal enema was given. We did not use peridural catheters, central venous catheters and urinary catheters. In 1 patient with an intraoperatively extense filling of the urinary bladder, we placed a suprapubic urinary catheter under laparoscopic control.

### 2.2. Operative Technique: Single-Port Laparoscopic Reversal of Hartmann's Procedure

**Figure 1:**
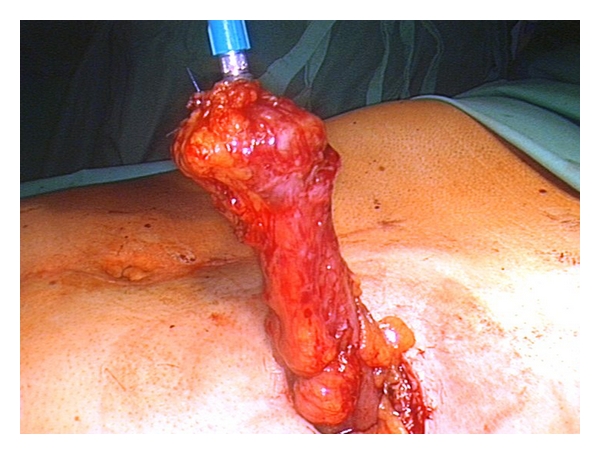
Colon descendens armed with the anvil.

The operation always started with the preparation of the colostomy. The stoma was excided and armed with clamps. After circular preparation in the subcutaneous tissue and in the fascial layer, the mobilized bowel was pulled out of the abdomen. A purse string clamp was placed 1-2 cm under the end of the bowel while the aboral portion was resected. The anvil of the circular stapler (28 mm diameter or bigger) was fixed by closing the purse string suture (Figure 1). 

The bowel was reponed into the abdominal cavity after dissecting local adhesions. 

**Figure 2:**
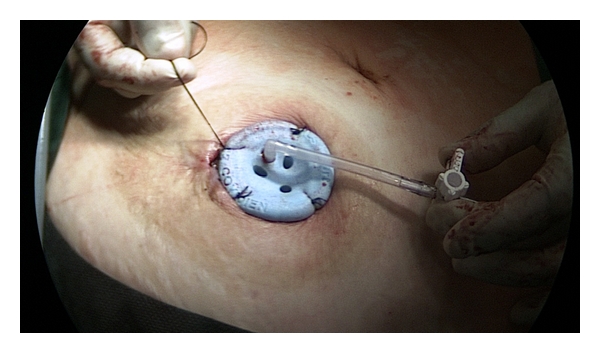
Placement of the single-port trocar at stomal side.

One special single port trocar with three instrument channels and one extra gas supply (SILS Port by Covidien) was introduced at the stoma site. To prevent dislocation, we fixed it to the wound with sutures (Figure 2).

After establishing the pneumoperitoneum, we introduced a special laparoscope with turnable lens and performed a diagnostic laparoscopy. By bringing the patient into anti-Trendelenburg position, the lower abdomen could be well visualized. 

**Figure 3:**
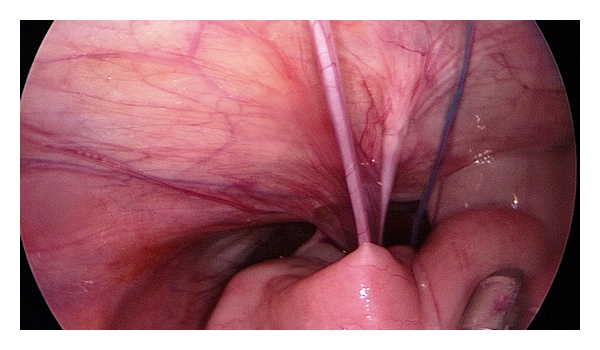
Adhesions in the lower abdomen.

As expected, we regularly found small bowel adhesions in the lower quadrants of the abdomen (Figure 3). 

**Figure 4:**
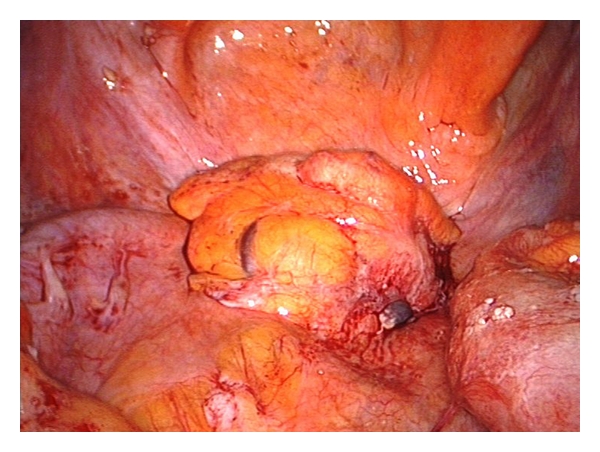
Scar tissue on the rectal stump.

Two 5 mm working trocars were used in the SILS port for dissector and mechanical or ultrasonic scissors. Close to the bowel, we only used a pair of mechanical scissors to prevent thermic damage to the bowel. When bleedings occurred, we used a bipolar clamp for coagulation. Adherent small bowel loops were gently pulled out of the small pelvis while dissecting the interenteric adhesions. The direct visualization of the rectal stump was sometimes difficult due to scar tissue in the pelvic floor (Figure 4). 

**Figure 5:**
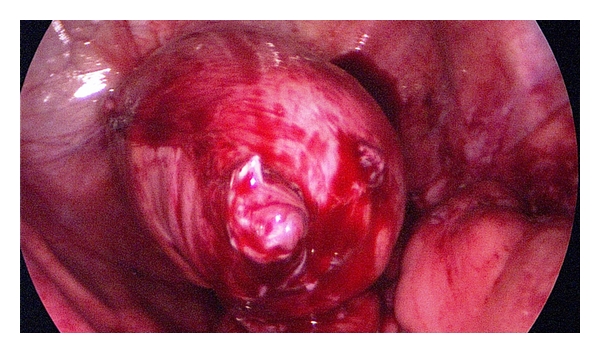
Rectal stump with 31 mm bougie.

By introducing a bougie via the anus, the rectal stump could be identified (Figure 5).

**Figure 6:**
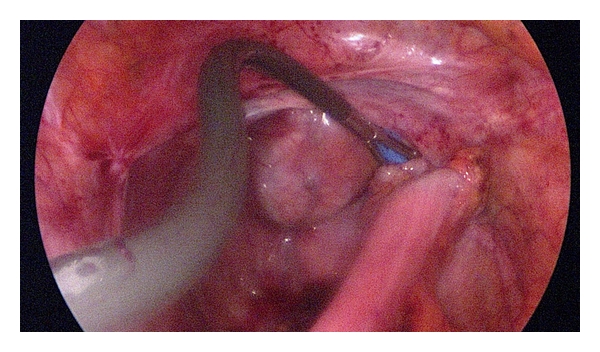
Length control of the colon descendens.

The oral part of the bowel with the anvil should be long enough to be brought to the pelvic floor without any tension (Figure 6). 

**Figure 7:**
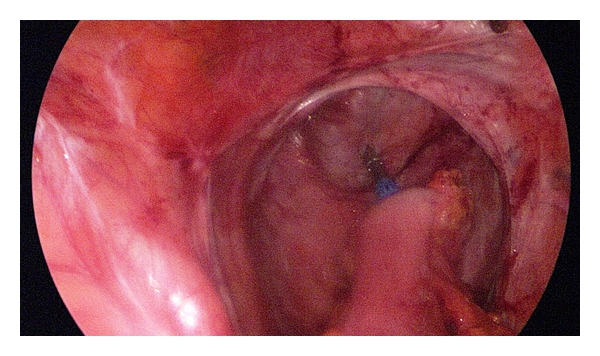
Connecting the anvil with the circular stapler.

**Figure 8:**
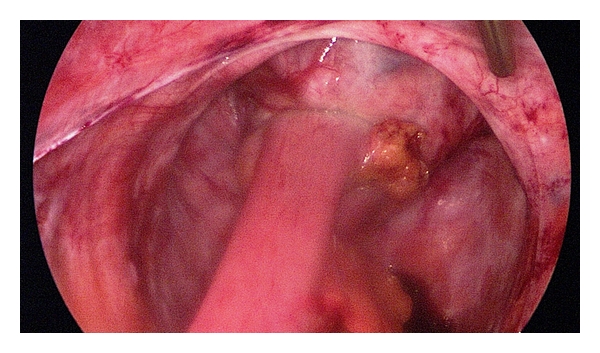
Circular anastomosis (CEEA 31).

Otherwise mobilisation of the left curvature is necessary. The circular stapler was transanally pushed to the top of the rectal stump, and the spike of the stapler should come out in the middle of the rectal stump, preferably close to the stapler line of the primary resection. After connecting the anvil, the stapler was closed and fired. (Figures 7 and 8). 

The stapler was then opened and removed transanally. To test the sufficiency of the anastomosis, the small pelvis was filled with saline solution. Air was pushed into the rectum via a transanal tube. If there were no air bubbles to be seen, the anastomosis had no leakage.

### 2.3. Postoperative Treatment

If there were no intraoperative complications, the patients were brought to the wake-up unit. They were allowed to drink free fluid on the day of surgery. On the first postoperative day they got soups, after the first stool normal food. The patients were discharged after 4–8 (*∅* 6.4) postoperative days.

## 3. Results

### 3.1. Patient Distribution

The youngest patient was 36 years old, and the oldest was 84 years old (average 60.4 years). 5 patients (63%) were females which outnumbered the 3 male patients. The BMI (body mass index) ranged from 24 to 38 (average 30.0). 

The intra- and postoperative results of the single-port laparoscopic reversal after Hartmann's procedure are shown in Tables 1 and 2.

Except of one superficial wound complication, the postoperative course was uncomplicated in all patients.

The patients were clinically examined after 14 and 30 days. For all patients, the postoperative followup was more than 30 days after the operation.

## 4. Discussion

Laparoscopic reversal after discontinuity colonic resection is technical demanding using 3-4 ports or one single-port device. Intraoperative difficulties are caused by existing adhesions in the lower abdomen. In our 8 patients, the rate of complications after single-port was very low. The relatively short operating time can be explained by our special technique.

We directly start with the mobilization of the stoma. In our technique, the primary preparing of the afferent loop saves time and allows an easy placement of the single-port device into the stoma incision [1]. There is no risk of bowel injury during the access to the abdomen. Even after primary conventional operation, single-port access through the stoma site is unproblematic. 

In literature, there are some single-center publications with relatively small groups of patients and varying operation techniques—open, laparoscopic with 3-4 trocars, or laparoscopically assisted operations. One author describes the reversal through the stomal side with manually adhesiolysis and manually controlled anastomosis [2]. In open surgery, Kunin et al. [3] described a morbidity of 47,8% and a mortality of 4,3% after reversal of Hartmann's operation. The rate of secondary anastomosis ranged from 7,1% (colonic cancer patients) to 65,4% (patients with complicated sigmoid diverticulitis). Keck et al. [4] performed the reversal in 52% of the patients (83% with diverticular disease). He found a complication rate of 26% and a mortality of 2%. Oomen et al. [5, 6] had a 3,1% mortality and a 38,5% morbidity in 63 patients, and Griffa et al. [7] reported 0% mortality and 37.5% postoperative complications after 32 reversals of Hartmann's procedure. He came to the conclusion that Hartmann's procedure should be used when patients are unsuitable to a one-step treatment because of their poor general and local conditions. Aydin et al. [8] reported that Hartmann's reversal was associated with a high prevalence of postoperative adverse events compared to primary resection and anastomosis. Dumont et al. [9] described an intestinal continuity restoration rate of 77% with a low mortality (0%), and morbidity (13%) in a selected group of patients.

In summary, literature shows a restoration rate of colonic continuity after Hartmann's operation between 7 and 77%. Mortality ranges between 0 and 15%, morbidity between 13 and 50%. 

With introduction of the laparoscopical reversal of Hartmann's procedure, the results became much better. Sosa et al. [10] attempted the laparoscopically assisted Hartmann's reversal in 18 patients with a conversion rate of 22,2%. He found a 0% mortality and a 14,3% morbidity. Macpherson et al. [11] had no conversion in twelve cases, 0% mortality and 25% complications. 

In 2010, Siddiqui et al. [12] published a first systematic review for open versus laparoscopic reversal of Hartmann's procedure. They concluded that laparoscopic procedure is safe has fewer complications and shorter hospital stays.

Other authors showed similar results (see Table 3).

These results show that the laparoscopic reversal after Hartmann's procedure is a safe method with a lower morbidity and mortality than after classical open reconstruction. 

Our first experiences with a single-port access show similar results. The avoidance of extra incisions, pain and possible complications by multiport access is one big advantage of single-port surgery combined with a better cosmetic result (Table 4). Access through the stomal side and manually preparation without laparoscopic instruments showed a conversion rate of 19% [2] and is therefore not recommended by us.

Another big advantage of minimizing the access trauma could be shown in the very short hospital stay. On average the patients could be discharged after 6.4 postoperative days. The small incision, almost no blood loss, and the short operating time could be the main reasons. In order to obtain statistical significance, further randomized studies are needed.

## 5. Conclusion

Laparoscopic reversal after Hartmann's procedure is a technical demanding and complex operation. The results of the actual literature and of our patients show a lower morbidity and mortality for the laparoscopic procedure compared to open operation. The high morbidity rates with up to 50% after conventional reversal could be reduced to less than 20% by minimally invasive surgery.

By using single-port access without any extra scar than the stoma incision, the access trauma and the rate of possible complications are lower compared to “conventional” laparoscopic surgery with 3-4 trocars. Primary dissection and preparation of the stoma before laparoscopy is very helpful, reduces the need of conversion and saves operating time. In difficult situations, there will be almost no time loss to use extra trocars or convert to open surgery.

We recommend the single-port laparoscopic reversal of Hartmann's procedure—independent of the kind of primary operation (open or laparoscopic).

## Figures and Tables

**Table 1:** Intraoperative results of single-port laparoscopic reversal.

	Patients (*n* = 8)	Percentage/Range
Conversion to open procedure	0	0%
Operating time	*∅*74 min	45–94 min
Bowel injury	0	0%
Blood loss	*∅*<50 mL	5–130 mL
Extra trocar needed*	1	12.5%

*failure of single-port attempt by 2nd trocar.

**Table 2:** Postoperative results of single-port reversal.

	Patients (*n* = 8)	Percentage/Range
Minor complications*	1	12.5%
Major complications	0	0%
Insufficiency of anastomosis	0	0%
Blood transfusion needed	0	0%
Reoperation	0	0%
Discharge from hospital	6.4 postoperative days	4–8

*1 superficial wound infection.

**Table 3:** Results after laparoscopic multiport reversal of Hartmann's procedure.

	Patients (*n*)	Conversion rate (%)	Complications, morbidity (%)	Mortality (%)
Sosa	18	22.2	14.3	0
Macpherson	12	0	25	0
Koehler*	19	11	18.8	0
Vacher*	38	15	23.5	2.6
Rosen	22	9	14	0
Carus*	28	17.9	17.9	0

*primary dissection and preparation of the stoma before laparoscopy.

**Table 4:** Comparison: multiport versus single-port reversal of Hartmann's procedure.

	Patients (*n*)	Conversion rate (%)	Complications, morbidity (%)	Mortality (%)
Range*	12–38	0–22.2	14–25	0–2.6
Carus**	8	0	12.5	0

*range: data from Table 3—multiport laparoscopic reversal.

**single-port laparoscopic reversal of Hartmann's procedure.
